# Solving an airport ground service task assignment problem with an exact algorithm

**DOI:** 10.1371/journal.pone.0279131

**Published:** 2022-12-22

**Authors:** Qiannan Tian, Jie Li, Guoxuan Huang, Wei Yuan

**Affiliations:** 1 Hubei Logistics Development Research Center, Hubei University of Economics, Wuhan, Hubei, China; 2 School of Management, Huazhong University of Science and Technology, Wuhan, Hubei, China; 3 School of Business Administration, Hubei University of Economics, Wuhan, Hubei, China; Chang Gung University, TAIWAN

## Abstract

In this paper, an airport ground service task assignment problem is studied. A task represents a service, which must be performed by one or multiple ground crew of a shift with required qualification/proficiency within a prescribed time period. For every assigned task, define “task priority” times “task duration” as the “benefit” generated. The objective is to maximize the summation of “benefit” for all the assigned tasks. The problem is modeled as an integer linear programming problem with mathematical formulation. A branch-and-price algorithm is proposed for solving the problem instances to optimality. To expedite the column generation process, an acceleration strategy is proposed. The computational results show that our proposed branch-and-price algorithm is capable of solving large-sized instances and the acceleration strategy is quite effective in reducing the computational time. Moreover, the impact of changing various characteristics of tasks and shifts on the performance of the algorithm is studied in detail with supporting computational experiments. In particular, the impact of reducing the qualifications is significant with 20.82% improvement in the objective value.

## 1 Introduction

According to a McKinsey study, the airline industry’s total revenue was $328 billion in 2020, only about 40% of the previous year, due to the impact of COVID-19. The study expects the industry to be smaller in the coming years, with traffic volumes not expected to return to 2019 levels until 2024 [1]. Global airlines carried more than 440.55 million passengers and 7.31 million tons of freight in 2021. Providing these services directly creates 5.1 million jobs in the air transport industry and contributes $439.5 billion to global GDP. As one of the important components of air transport, the airport ground handling service is crucial to the turnaround of aircrafts and for the smooth running of daily operations of an airport [2]. The airport ground handling services can be divided into two basic types based on their locations: terminal and ramp. Terminal handlings are performed inside the airport terminal buildings and directly concern the passengers. Providing check-in counter services, managing VIP customer services and disabled passengers, staffing the transfer counters, customer service counters and airline lounges are a few examples. On the other hand, ramp activities take place at the aircraft parking position between the time the aircraft arrives at a terminal gate and the time it departs on its next flight [3, 4]. Some examples of ramp activities include marshaling the aircraft, offloading and loading of passengers and baggage, catering, providing ground power/air conditioning to the aircraft, deicing and push-back tractor services. Speed, efficiency and accuracy are important in ground handling services in order to minimize the turnaround time [5]. Over the years, owing to globalization and liberalization of economies, the number of third-party ground handlers has grown substantially resulting in better service, lower price and higher operational efficiency [6].

This research is motivated by a consulting project for an airport ground handling services provider interested in optimizing its terminal handling services. Each day, the service handler has hundreds of tasks pending to be assigned to a shift pool (a group of workers). A task represents a service (differentiated by different task types), which must be performed by one or multiple ground crew with required qualification/proficiency within a prescribed time period. A sample qualification could be a language requirement or mastery of a certain airline’s check-in system. When a qualification requirement is given, its corresponding proficiency requirement, on a scale of 1—5, is also provided. For instance, a task might require the ground crew to speak German language (i.e., qualification) fluently (i.e., proficiency). Moreover, each task is assigned a priority value that is derived from several factors and revised on a periodic basis. Some of the factors include, the impact of the task on passenger comfort, adherence to the stipulated frequency of execution in a given timeframe (e.g. rest rooms are to be cleaned six times in a day, vending machines are to be serviced once in fortnight), relationship between the task and perceived service quality and customer satisfaction, so on and so forth. A shift is a period of time for one or multiple workers of exactly the same qualifications to work together on exactly the same tasks. A shift gives the starting and ending time and a list of the qualification/ proficiency pairs associated with one or multiple ground crew. For example, a task may need 3 crew members of identical qualifications to work together for its execution. Also, it is not uncommon to expect ground crew to be multi-skilled. For instance, under some circumstances, the ground crew is required to speak two different languages (with required proficiency level) and master three airlines’ check-in systems. The following Tables 1 and 2 provide sample information about different tasks and shifts.

**Table 1:** Sample tasks with their details.

Task ID*	Description	Start time	End time	Priority	Minimal proficiency
1586	Managing International flight boarding gates—Essential knowledge of spoken English	235	370	3	Fluent
1531	Preventive maintenance of coffee vending machines at the lounge	145	225	2	Strong
2051	Aircraft cleaning	100	130	5	Expert
1202	Cleaning/maintaining of VIP lounge	200	345	4	Above average
1787	SUV Pick-up of pilot & co-pilot	300	320	2	Experienced

*: ID of the qualification to perform task, belongs to real number

**Table 2:** Sample shifts with their details.

**Shift No: 1**	**Start Time: 240**	**End Time: 720**
**Qualifications with ID**	**Proficiency**	
Management ability(1511)	Strong	
Oral English level(1586)	Fluent	
**Shift No: 2**	**Start Time: 170**	**End Time: 600**
**Qualifications with ID**	**Proficiency**	
VIP customer service(2048)	Strong	
Driving of SUV(1787)	Experienced	
**Shift No: 3**	**Start Time: 440**	**End Time: 1040**
**Qualifications with ID**	**Proficiency**	
Aircraft cleaning(2051)	Beginner	
Pushback tractor operation(1800)	Expert	

Shifts are limited in number compared to the tasks, and hence not all the tasks need to be completed. Relatively important tasks are often given high priority, such as providing VIP services to VIP customers usually carries very high priority but may not consume much time. In contrast, tasks such as preventive maintenance of vending machines, maintaining clean and tidy waiting halls and restrooms consume lot of labor hours but may get assigned relatively lower priority. These low priority tasks serve a relatively large number of people. In addition, the low priority tasks are also important from airport service quality/revenue and passenger comfort perspectives. Therefore, with a view to strike a balance amongst all the tasks, we define “benefit” associated with every assigned task as the product of its ‘priority’ and ‘duration’. The objective is to maximize the sum of “benefits” of assigned tasks. The problem of assigning different tasks to shifts is usually done in two phases. The first phase, which this work addresses, aims at an optimal preliminary assignment. In the second phase, careful examination of the unassigned/left over tasks is carried out to assess if any corrective measures, such as changing the start/end time or revising the priority values, are to be undertaken to manually refine the assignment further. It is noteworthy that, in reality, certain tasks that are directly related to the safety of the aircraft and are to be performed mandatorily are usually assigned very high priority values so as to include them in the first phase itself. A task can be assigned to a shift only if the following constraints are satisfied:

- The shift possesses all the skills required to perform the task in the required proficiency level.
- The starting/end time of a shift should be prior to/later than the starting/end time of the task.
- The same shift cannot be assigned two tasks if there is not enough traveling time between them. This is particularly important at large airports where the tasks are geographically dispersed (i.e., different terminals, boarding gates, checking-in points).
- The shift performs only one task at a time.
- The shift completes all the assigned tasks in entirety.
- The same task is not simultaneously executed by two shifts.

Until recently, before the deployment of this algorithm, the service provider used to assign the tasks to shifts manually based on the planners’ intuitive skills and their past work experience. This practice was resulting in heavy workloads for the planners and sub-optimal resource utilization plans. Moreover, with the increase in the air traffic day-by-day resulting in the increase of both the number of tasks and shifts, it was becoming impractical to assign tasks manually within short time. The scheduling problem of ground service crew traveling from one aircraft parking place to another, which is a subset of the task assignment problem, is shown to be equivalent to the vehicle routing problem with time windows (VRPTW) for multiple non-identical vehicles [2]. It is a well-known fact that the VRPTW is a *NP*-hard problem [7] and hence the problem being studied in this work also falls under the same category. In fact, this combinatorial optimization problem of assigning various tasks to shifts is similar to assignment problems that arise in parallel and distributed computing [8, 9].

The literature available on airport ground service task assignment problem is very sparse. Given the cost implications associated with the problem, this research work aims at solving real-life problem instances of the task assignment problem with an exact algorithm, i.e., branch-and-price algorithm. The problem is formulated as a network optimization problem with mathematical formulation. In order to solve the problem, the model is decomposed into a master problem and a sub-problem using Dantzig-Wolfe decomposition methodology. The sub-problem is solved by a label setting algorithm to find columns with positive reduced costs. The column generation solves the relaxed master problem and thus cannot guarantee the integrality of the solution. Therefore, we implement a branch and bound algorithm to ensure the integrality of variables. To efficiently solve the sub-problem, we propose a hybrid accelerating strategy which combines the label setting algorithm with a heuristic procedure.

The remainder of this paper is organized as follows. Section 2 presents a review of relevant studies. In Section 3, mathematical formulation for the task assignment problem is presented. In Section 4, the formulated problem is decomposed using Dantzig-Wolfe decomposition method and then the branch-and-price algorithm is presented. Results of the computational experiments are presented in Section 5. Conclusions, contributions of this work and possible future research directions are discussed in Section 6.

## 2 Literature review

In this Section, we review the literature pertaining to the task assignment problem as well as related problems that are relevant in the context of ground handling services. By and large, the staff assignment and scheduling problem is solved in three steps [2, 10]. The first step deals with specifying a sequence of days of work or days-off for each crew, which is constrained by the crew’s working contract or collective agreements. The second step is shift scheduling, which is to select shifts from a shift pool based on workforce demand or shift duties. The last step is crew assignment/rostering, which consists of assigning the selected shifts to specific tasks with the intent of maximizing the utility. Ip et al. [2] study the optimization problem of scheduling the services of aircraft ground service crew where the traveling time between different parking locations of aircrafts is significant. A genetic algorithm with a hybrid encoding scheme is proposed to solve the problem. Soukour et al. [10] consider the problem of scheduling/rostering the airport security staff. A memetic algorithm that unifies an evolutionary algorithm with local search techniques is proposed. Chu [11] develop a Goal programming based approach for an integrated crew-duties assignment problem for baggage handling section staff at the Hong Kong International Airport.

Since the fees payable to the ground crew constitute the second highest source of cost for an airline, the first being fuel costs, optimized crew pairing is crucial to cost saving. Generally, crew pairing problem consists of two procedures: crew pairing generation and crew rostering. Muter et al. [12] study a robust airline crew pairing problem where the airline is faced with the challenge of adding extra flights at short notice while minimizing the disruptions to the original plans. The authors propose a column generation based solution technique for solving the problem. Zeren and Özkol [13] also use column generation to solve a large-scale airline crew pairing problem. Quesnel et al. [14] study an extension of the crew pairing problem, which includes additional constraints on the total work time of each crew. They propose a retrospective branching heuristic that outperforms other solution methodologies. Zeng et al. [15] consider the airport ground workforce planning, whose objective is to minimize the weighted sum of numbers of employees in a tour scheduling model with respect to a set of scheduling rules.

The problem of assigning tasks to shifts that match all the requirements while maximizing the “benefits” can be considered as a special case of Generalized Assignment problem (GAP) that has numerous applications in the aviation industry and distributed computing systems [16]. Other similar application of the assignment problem can be found in assigning items to storage locations in a warehouse [17] and assigning aircrafts to terminal gates [18]. Lai et al. [19] study the task assignment problem in a distributed computing environment that aims to assign multiple application tasks to a set of heterogeneous processors with the objective of minimizing the total computational cost. The application tasks/processors are similar to tasks/shifts in the task assignment problem. The authors propose an Entropic simplified swarm optimization procedure to solve the problem. Yedidsion and Shabtay [20] study a resource dependent assignment problem, where the cost of assignment is resource specific. After proving the computational complexity of the problem to be NP-hard, the authors develop an approximation algorithm for solving the problem.

One of the straightforward ways to solve the task assignment problem is by exhaustive enumeration. Though this approach may work for small problem instances, it will be quite impractical for real-life large instances. For instance, the airport ground handling service provider who provided us with the data routinely deals with tasks and shifts that run into hundreds. One of the ways to circumvent this enumeration is to use column generation technique that implicitly considers all the possibilities while keeping the number of variables at an acceptable level [14, 21]. Column generation has been successfully applied to solve a variety of routing and scheduling problems, such as airline crew pairing [12–14, 22], aircraft sequencing problem [23–25]; pickup and delivery problem with time windows [26–30], vehicle routing problem [31–34], vehicle routing problem with time windows (VRPTW) [7, 21, 35–37] and the variant of VRPTW [38, 39]. There are some similarities between the task assignment problem and the vehicle routing problem with time windows (VRPTW). In the context of vehicle routing, shifts and tasks are analogous to vehicles and customers respectively. However, since task and shift have multiple attributes, the constraints considered and the establishment of mathematical programming model are more complicated. Hence, a straight forward adaptation of VRPTW solution methodologies cannot be used to solve the task assignment problem in this paper. In particular, the design of Label structure, extension functions and dominance rule of Sub-problem is not the same as before.

Given the status of literature pertaining to the task assignment problem, we opine that the problem is new and has not received adequate attention from the researchers. In this paper, we intend to develop an exact algorithm that can solve real-life problem instances within acceptable computational time (typically, less than 3600 seconds). Moreover, an accelerating strategy is proposed to expedite the solving process.

## 3 Mathematical formulation

In this section, the task assignment problem is modeled as an integer linear programming problem by way of mathematical formulation. First, *T* = {0, 1, 2, …, *N*} denotes the set of tasks, where the number of tasks is *N* + 1. In particular, 0 represents the dummy task. *S* = {1, 2, …, *M*} represents the set of shifts, where the number of shifts is *M*. A directed graph *G* = (*T*, *E*) is defined to formulate the considered problem, where *E* = {(*i*, *j*):*i*, *j* ∈ *T*} is the set of feasible arcs and each arc (*i*, *j*)∈*E* means that task *j* is performed immediately after task *i*. As the problem is solved on a daily basis, the planning horizon is restricted to one day only. Notations for the model are defined in Table 3.

**Table 3:** Notations.

**Parameters**	
$D_{i}^{a}$	Start time of task *i*, *i* ∈ *T*
$D_{i}^{e}$	End time of task *i*, *i* ∈ *T*
$T_{i}^{t}$	Type of task *i*, *i* ∈ *T*
$T_{i}^{p}$	Priority of task *i*, *i* ∈ *T*
$T_{i}^{q}$	ID of the qualification requirement to perform task i, $i\in T,\;T_{i}^{q}\in R^{+}$
$T_{i}^{pro}$	Minimal proficiency required to perform task i, $T_{i}^{pro}=\left\{0,1,2,3,4,5\right\}\;$
$S_{s}^{a}$	Start time of shift *s*, *s* ∈ *S*
$S_{s}^{e}$	End time of shift *s*, *s* ∈ *S*
$S_{sn}^{q}$	ID’s of ‘*n*’ qualification possessed by shift *s* and *n* ranges between 1 and 9, $s\in S,\;n\in \left\{1,2,...,9\right\},\;S_{sn}^{q}\in R^{+}\;$
$S_{sn}^{pro}$	Measures the proficiencies of ‘n’ qualifications possessed by shift *s* and n ranges from 1 to 9,$s\in S,\;\;n\in \left\{1,2,...,9\right\},\;S_{sn}^{pro}=\{0,1,2,3,4,5\}$
*d*_*ij*_	Distance between the places where task *i* and *j*, *i*, *j* ∈ *T* are performed. The distance is valued by time
*c*_*ijs*_	Generated benefit when tasks *i* and *j* are performed by shift *s*. It is equal to $(D_{i}^{e}-D_{i}^{a})\times T_{i}^{p}+(D_{j}^{e}-D_{j}^{a})\times T_{j}^{p},\;i,j\in T$. Task i is performed prior to task j
$\bar{M}$	Large non-negative integer
**Decision Variables**	
*x*_*ijs*_	Equals to 1 if shift *s* ∈ *S* performs task *j* ∈ *T* immediately after task *i* ∈ *T* and 0 otherwise.

The arc-flow formulation of the task assignment problem is modeled as follows:

$$\begin{array}{cc} Maximize\;Z & =\frac{1}{2}\sum_{s\in S}\sum_{i\in T}\sum_{j\in T}c_{ijs}x_{ijs}, \end{array}$$ (1)

$$\begin{array}{cc} s.t.\;\sum_{i\in T}\;x_{0is} & =1,s\in S, \end{array}$$ (2)

$$\begin{array}{cc} \sum_{i\in T}\;x_{i0s} & =1,s\in S, \end{array}$$ (3)

$$\begin{array}{cc} \sum_{s\in S}\;x_{ijs} & \leq 1,(i,j)\in E, \end{array}$$ (4)

$$\begin{array}{cc} \sum_{i\in T,i\neq h}\;x_{ihs} & -\sum_{j\in \text{T},j\neq h}\;x_{hjs}=0,h\in T,s\in S, \end{array}$$ (5)

$$\begin{array}{cc} \sum_{i\in T,i\neq 0}\;x_{iis} & =0,s\in S, \end{array}$$ (6)

$$\begin{array}{cc} S_{sn}^{q}\sum_{j\in T,j\neq i}\;x_{ijs} & =T_{i}^{q}\sum_{j\in T,j\neq i}\;x_{ijs},i\in T,\;i\neq 0,\;s\in S,\;n\in \{1,2,\ldots ,9\}, \end{array}$$ (7)

$$\begin{array}{cc} T_{i}^{pro}\sum_{j\in T,j\neq i}\;x_{ijs} & \leq S_{sn}^{pro}\sum_{j\in T,j\neq i}\;x_{ijs},i\in T,\;i\neq 0,\;s\in S,\;n\in \{1,2,\ldots ,9\}, \end{array}$$ (8)

$$\begin{array}{cc} S_{s}^{a}-D_{i}^{a} & \leq (1-x_{ijs})\bar{M},\left(i,j\right)\in E,i\neq j,i\neq 0,s\in S, \end{array}$$ (9)

$$\begin{array}{cc} D_{i}^{e}-\;\;\;\;\;\;\;S_{s}^{e} & \leq (1-x_{ijs})\bar{M},\left(i,j\right)\in E,i\neq j,i\neq 0,s\in S, \end{array}$$ (10)

$$\begin{array}{cc} D_{i}^{e}+d_{ij}-D_{j}^{a} & \leq (1-x_{ijs})\bar{M},\left(i,j\right)\in E,i\neq j,i\neq 0,j\neq 0,s\in S, \end{array}$$ (11)

$$\begin{array}{cc} \sum_{i\in T⋃\;\;\;\{\;\;\;0\;\;\;\}\;\;\;}\;\sum_{j\in T,j\neq i}\;\sum_{s\in S}\;x_{ijs} & \leq N, \end{array}$$ (12)

$$\begin{array}{cc} \sum_{i\in T}\;\sum_{s\in S}\;x_{0is} & \leq M, \end{array}$$ (13)

$$\begin{array}{cc} x_{ijs} & \in \{0,1\},\;\text{(}i,j)\in E,s\in S. \end{array}$$ (14)

The above formulation is an integer linear programming (ILP) model. The objective function (1) is to maximize the sum of all the generated benefits. Since the benefit of each performed task is calculated twice, the coefficient 1/2 needs to be added to the objective function. Constraint (2) indicates that every shift starts from task 0, which represents a dummy task. Constraint (3) indicates that every shift will return to task 0 at the end. Constraint (4) implies that any task is performed by one shift at most. Constraint (5) represents the flow conservation constraint. Constraint (6) ensures that a task is not allowed to be performed continuously by the same shift. Constraint (7) means when a task is performed by a shift, the required qualification to perform the task must be met. Constraint (8) means the corresponding qualification proficiency of the shift should not be lower than that required by the task. On similar lines, Constraints (9) & (10) ensure the compatibility between tasks and assigned shifts in terms of start and end times. Constraint (11) indicates that when two tasks are performed by the same shift, the distance constraint is explicitly satisfied. All the distance matrices considered in this study strictly meet the triangular inequality. Constraint (12) represents the total number of tasks performed cannot exceed the available number. Constraint (13) requires that the number of utilized shifts cannot exceed the available number. Constraint (14) is the binary requirement on variable *x*_*ijs*_.

Preliminary tests conducted to solve some randomly generated problem instances using the CPLEX solver were successful but only for small-sized instances. For example, when the size of instance is T43-s13, the running time is less than 1 minute. However, when the size of instance is greater than T140-s42, CPLEX can’t solve the instance in the specified time (3600s). Therefore, a better approach is needed to solve large-sized instances within the stipulated computational time. Branch-and-price algorithm is one such exact method that gives optimal solutions by generating columns on a branch-and-bound tree. A detailed discussion on the branch-and-price algorithm is presented in Section 4.

## 4 Enhanced branch-and-price algorithm

The branch-and-price algorithm is implemented in a branch and bound framework, where column generation procedure is used to solve the LP relaxations at each search tree node. Applying Dantzig-Wolfe Decomposition reformulates integer linear programming model to form a master problem and a sub-problem (Dantzig and Wolfe [40]). Instead of explicitly enumerating all the columns (variables), the master problem considers only a subset of columns and relaxes the integrality restrictions on the variables, which is referred to as a restricted linear master problem (RLMP). Column generation algorithm obtains optimal solution by iterating between the RLMP (restricted linear master problem) and the sub-problem. Every time when an iteration starts, the primal simplex algorithm is used to solve the linearly relaxed master problem. By solving the linearly relaxed master problem, values of the dual variables are obtained, which will be used in the objective function of the sub-problem. The sub-problem is solved by a label setting algorithm to find columns (variables) with positively increased benefits, which will, then, be added to the master problem. Since the relaxed linear master problem may return a fractional solution, the column generation technique will be embedded into a branch-and-bound search tree. This entire process is known as the branch-and-price algorithm in the literature [22, 41, 42].

Before applying the Dantzig-Wolfe Decomposition principle, the feasible domain of the sub-problem has to be defined. Let *D*^*s*^ = {(*x*^*s*^, *S*^*a*^, *S*^*q*^, *S*^*pro*^) | (*x*^*s*^, *S*^*a*^, *S*^*q*^, *S*^*pro*^), ∀ *s* ∈ *S*} be the feasible domain of the sub-problem that satisfies constraints (2)—(3) and (5)—(11). By substituting (*x*^*s*^, *S*^*a*^, *S*^*q*^, *S*^*pro*^), the convex combination of the extreme points of *D*^*s*^ for all *s* ∈ *S*, in the rest of the mathematical model, the master problem and the sub-problem of the task assignment problem are formulated as follows.

### 4.1 Master problem

It is straightforward to decompose the MIP formulation based on shifts to derive a master problem that involves deciding the best routes for all the shifts. The following additional notations in Table 4 are defined before presenting the master problem.

**Table 4:** Notations for master problem.

Parameters	
*P*	Set of all feasible routes (composed by the set of tasks) for shifts. A route *r* ∈ *P* (which is represented by one column) can be written as *r* = {*t*_0_, *t*_1_, *t*_2_, ⋯, *t*_*K*_, *t*_*K* + 1_}, such that each arc (*t*_*k*_, *t*_*k* + 1_) belongs to *E*, *t*_0_ = 0 and *t*_*K* + 1_ = 0. Route ‘0’ represents the empty route.
*P*_*s*_	Set of the feasible routes of shift *s* ∈ *S*, with ⋃_*s* ∈ *S*_ *P*_*s*_ = *P*
$c_{r}^{s}=\sum_{i,j\in r}\;c_{ijs}$	The generated benefit of route *r* ∈ *P*_*s*_
$\delta_{ir}^{s}$	Equals to 1 if shift *s* ∈ *S* executes task *i* on route *r* ∈ *P*_*s*_, and 0 otherwise;
$x_{r}^{s}$	Binary decision variable, which equals to 1 if route *r* ∈ *P*_*s*_ of shift *s* ∈ *S* is selected in the final solution and 0 otherwise.
*H*	Non-negative value indicating the total number of tasks performed by the given shifts

With these notations, the master problem can be remodeled as following set partitioning formulation:

$$\begin{array}{cc} Maximize\;Z & =\frac{1}{2}\sum_{s\in S}\;\sum_{r\in P_{s}}\;c_{r}^{s}x_{r}^{s}, \end{array}$$ (15)

$$\begin{array}{cc} s.t.\;\sum_{s\in S}\;\sum_{r\in P_{s}}\;\delta_{ir}^{s}x_{r}^{s} & \leq 1,\forall i\in T, \end{array}$$ (16)

$$\begin{array}{cc} \sum_{s\in S}\;\;\sum_{r\in P_{s}}\;x_{r}^{s} & \leq M, \end{array}$$ (17)

$$\begin{array}{cc} \sum_{i\in T}\;\sum_{s\in S}\;\sum_{r\in P_{s}}\;\delta_{ir}^{s}x_{r}^{s} & \leq N, \end{array}$$ (18)

$$\begin{array}{cc} x_{r}^{s} & \in \{0,1\},\forall s\in S,r\in P_{s}. \end{array}$$ (19)

The objective function (15) aims at maximizing the sum of the total benefits generated. Constraint (16) ensures that every task can be executed by at most one shift. Constraint (17) ensures that the number of shifts that can be used to perform the tasks cannot exceed its total given number. Constraint (18) represents the total number of performed tasks cannot exceed the available number and Constraint (19) indicates the binary nature of the decision variable.

The structure of the master problem closely resembles that of the typical set partitioning problem. In the incidence matrix *A* shown below, the rows of the matrix represent the tasks and the columns represent the shifts. The entries {0, 1} represent the assignment of tasks to shifts.

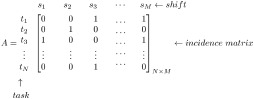


The master problem (MP) is an integer programming (IP) model. By dropping the integrality requirements on $x_{r}^{s}$, a linear relaxation of the MP can be obtained. The aim is to obtain the optimal solution of the relaxed master problem, which is the upper bound of its corresponding branch-and-bound node. The set partitioning problem is a proven *NP*-complete problem and computationally intractable for most of the real-life applications. As most of the columns will be non-basic and have their corresponding variable value equal to 0 in the optimal solution, the restricted linear master problem (RLMP) that only considers a subset of the columns is solved. Promising columns are dynamically appended to the RLMP. The branching procedure is activated if there are no more columns to be added and the integrality conditions are not satisfied.

### 4.2 Sub-problem

Any column (variable) with positive *reduced cost* (being a maximization problem) is a candidate to enter the basis. Hence the sub-problem is to find a variable $x_{r}^{s}$ having a positive *reduced cost*, whose generation is in connection with the dual variables of the RLMP. We define the following addition notation to deal with the dual variables associated with the constraints.

*π*_*i*_: Dual variables of constraint (16) for task *i* ∈ *T*;

λ: Dual variable of constraint (17);

*β*: Dual variable of constraint (18);

${\bar{c}}_{ijs}$ : Positive reduced cost when tasks *i*, *j* ∈ *T* are performed by shift *s*;

${\bar{c}}_{r}^{s}=\sum_{i,j\in r}\;{\bar{c}}_{ijs}$ : Positive reduced costs when tasks *i*, *j* ∈ *r*, *r* ∈ *P*_*s*_ are performed by shift *s* ∈ *S*.

With these notations, ${\bar{c}}_{r}^{s}$ is given by

$$\begin{array}{c} {\bar{c}}_{r}^{s}=\sum_{i,j\in r}\;{\bar{c}}_{ijs}=\sum_{i\in T}\;\delta_{ir}^{s}\pi_{i}+\lambda +\sum_{i\in T}\;\delta_{ir}^{s}\beta -c_{r}^{s}, \end{array}$$ (20)

where

$$\begin{array}{c} c_{r}^{s}=\sum_{i,j\in r}\;c_{ijs}. \end{array}$$ (21)

The criterion for satisfying the optimality condition of the master problem is:

$$\begin{array}{c} \alpha_{r}^{s}=\sum_{i\in r}\;\delta_{ir}^{s}\pi_{i}+\lambda +\sum_{i\in r}\;\delta_{ir}^{s}\beta -c_{r}^{s}\geq 0. \end{array}$$ (22)

That is to say, if the minimum test number satisfies (23), the master problem is optimal.

$$\begin{array}{c} \alpha^{*}={\text{min}}_{r\in P}\{\alpha_{r}^{s}=\sum_{i\in r}\;\delta_{ir}^{s}\pi_{i}+\lambda +\sum_{i\in r}\;\delta_{ir}^{s}\beta -c_{r}^{s}\geq 0\}, \end{array}$$ (23)

Therefore, the sub-problem can be reformulated as:

$$\begin{array}{c} \text{min}\;\alpha_{r}^{s}, \end{array}$$ (24)

$$\begin{array}{c} s.t.\;r\in P. \end{array}$$ (25)

In order to obtain high quality columns, the sub-problem can be described as a weighted constrained shortest path problem. Therefore, the sub-problem (aimed at finding a feasible route with the highest positive *reduced cost*) can be described by the following mathematical model:

$$\begin{array}{cc} Minimize\;Z & =\sum_{i\in T}\;\delta_{ir}^{s}\pi_{i}+\lambda +\sum_{i\in T}\;\delta_{ir}^{s}\beta -\sum_{i,j\in r}\;c_{ijs}x_{ijs}, \end{array}$$ (26)

$$\begin{array}{cc} s.t.\;\sum_{i\in T}\;x_{0is} & =1, \end{array}$$ (27)

$$\begin{array}{cc} \sum_{i\in T}\;x_{i0s} & =1, \end{array}$$ (28)

$$\begin{array}{cc} \sum_{i\in T,i\neq h}\;x_{ihs} & -\sum_{j\in T,j\neq h}\;x_{hjs}=0,h\in T, \end{array}$$ (29)

$$\begin{array}{cc} \sum_{i\in T,i\neq 0}\;x_{iis} & =0, \end{array}$$ (30)

$$\begin{array}{cc} S_{sn}^{q}\sum_{j\in T,j\neq i}\;x_{ijs} & =T_{i}^{q}\sum_{j\in T,j\neq i}\;x_{ijs},i\in T,\;i\neq 0, \end{array}$$ (31)

$$\begin{array}{cc} T_{i}^{pro}\sum_{j\in T,j\neq i}\;x_{ijs} & \leq S_{sn}^{pro}\sum_{j\in T,j\neq i}\;x_{ijs},i\in T,\;i\neq 0, \end{array}$$ (32)

$$\begin{array}{cc} S_{s}^{a}-D_{i}^{a} & \leq (1-x_{ijs})\bar{M},\left(i,j\right)\in E,i\neq j,i\neq 0, \end{array}$$ (33)

$$\begin{array}{cc} D_{i}^{e}-S_{s}^{e} & \leq (1-x_{ijs})\bar{M},\left(i,j\right)\in E,i\neq j,i\neq 0, \end{array}$$ (34)

$$\begin{array}{cc} D_{i}^{e}+d_{ij}-D_{j}^{a} & \leq (1-x_{ijs})\bar{M},\left(i,j\right)\in E,i\neq j,i\neq 0,j\neq 0, \end{array}$$ (35)

$$\begin{array}{cc} x_{ijs} & \in \{0,1\},\left(i,j\right)\in E. \end{array}$$ (36)

The objective function (26) is to minimize the positive reduced costs with respect to the dual variables of the RLMP for every *s* ∈ *S*. Constraints (27)-(36) construct a task assignment for every *s* ∈ *S*. The sub-problem is a variant of the elementary shortest path problem with resource constraints (ESPPRC) and therefore is *NP*-hard in the strong sense. This is considered as a main drawback of the branch-and-price algorithm in terms of computational efficiency, as it requires repetitive applications of an algorithm to find positive reduced cost columns.

### 4.3 Label setting algorithm for sub-problem

Label setting algorithm has been proved to be particularly efficient in solving ESPPRCs. A common practice to solve the ESPPRC is to develop a label setting algorithm based on dynamic programming [22, 43, 44]. In a label setting algorithm, a label traces the track of a partial path from source task 0 to task *i* ∈ *T* and also stores additional valuable information along with it. At each step, one label is selected and extended to all possible successive nodes. Some labels, that do not satisfy the resource constraints, will be discarded. In our label setting algorithm, both the label and the dominance rule have been modified to better suit the characteristics of our problem. The following sub-sections will provide finer details of the label setting algorithm.

**1**) **Label Structure**

Label structures for variants of the ESPPRC share some similarities with each other. Let label *L* be defined as $L=(C,TI,S_{}^{q},S^{pro},V^{t_{1}},\ldots ,V^{t_{N}})$, where *C* represents the positive reduced cost of the partial path; *TI*, time of the label; *S*^*q*^ and *S*^*pro*^ represent qualification and proficiency of shift, respectively; $V^{t_{i}}$, a binary variable, which indicates execution status of task *i* ∈ *T*.

**2**) **Extension Functions**

Starting from an initial label associated with source task 0, the algorithm extends towards all reachable adjacent nodes using extension functions. For a task *i* ∈ *T* whose resource time (start time, end time) is $\left[D_{i}^{a},D_{i}^{e}\right]$, *C*_*i*_ and *TI*_*i*_ respectively represent the reduced cost and start time elements of a label *L*_*i*_. A new label *L*_*j*_ with a reduced cost element $C_{j}=h_{ij}^{C}(C_{i})$ and a time element $TI_{j}=h_{ij}^{CSS}(TI_{i})$ can be generated by extending label *L*_*i*_ through arc (*i*, *j*), where the extension function $h_{ij}^{C}(C_{i})$ and $h_{ij}^{CSS}(TI_{i})$ are given by $h_{ij}^{C}(C)=C+{\bar{c}}_{ij}$ and $h_{ij}^{CSS}(TI)=TI_{j}=TI+d_{ij}$. If all the resource constraints are satisfied by the new label *L*_*j*_, then it is accepted as feasible. On the other hand, Lj would not be created if *TI*_*j*_ = (*TI*+ *d*_*ij*_) > *TS*_*j*_ or there exists a mismatch between task and shift in terms of qualifications or proficiency.

**3**) **Dominance Rule**

In the label extension process, one label will be extended to all reachable tasks, whereby, the number of labels will increase exponentially if all the labels are considered. This process turns out to be very inefficient even for small sized instances. To avoid such a circumstance, a dominance rule is employed to remove some unprofitable labels from the label pool. These unprofitable labels are dominated by other labels and will not affect the optimality of the sub-problem. Removing labels, whose extensions lead to infeasible routes, helps in expediting the label algorithm. Consider two labels $L_{i}^{1}=(C_{1},TI_{1},{S_{1}}^{q},{S_{1}}^{pro},{V_{1}}^{t_{1}},\cdots ,{V_{1}}^{t_{N}})$ and $L_{i}^{2}=(C_{2},TI_{2},{S_{2}}^{q},{S_{2}}^{pro},{V_{2}}^{t_{1}},\cdots ,{V_{2}}^{t_{N}})$, which represent two distinct partial paths ending at the same task *i* ∈ *T*. The following rules are considered to determine which label is dominant:

$$\begin{array}{c} \text{Positive}\;\text{reduced}\;\text{cost}\;\text{generated:}\;C_{2}\geq C_{1}, \end{array}$$ (37)

$$\begin{array}{c} \text{Time}\;\text{consumed:}\;TI_{2}\leq TI_{1}, \end{array}$$ (38)

$$\begin{array}{c} \text{Qualification:}\;{S_{2}}^{q}={S_{1}}^{q}, \end{array}$$ (39)

$$\begin{array}{c} \text{Proficiency}\;\text{possessed:}\;{S_{2}}^{pro}\leq {S_{1}}^{pro}, \end{array}$$ (40)

$$\begin{array}{c} \text{Visited}\;\text{tasks:}\;{V_{2}}^{t_{i}}\leq {V_{1}}^{t_{i}}. \end{array}$$ (41)

**Proposition 1**. *(Dominance Rule): Constraints* (37)—(41) *propose valid dominance rules*.

**Proof**. *If* *TI*_2_ ≤ *TI*_1_, ${S_{2}}^{q}={S_{1}}^{q}$ *and* ${V_{2}}^{t_{i}}\leq {V_{1}}^{t_{i}}$, *it indicates that the task that can be reached from* $L_{i}^{1}$ *can also be reached from* $L_{i}^{2}$ *by ensuring feasibility. Since* *TI*_2_ ≤ *TI*_1_, *it implies that any task executed after* $L_{i}^{1}$ *without violating time related constraints can also be executed after* $L_{i}^{2}$. *The positive reduced cost of a path is determined by its yielded benefit and the dual values of the executed tasks. So, every feasible extension of label* $L_{i}^{1}$ *is a feasible extension of label* $L_{i}^{2}$ *with an improvement. When a label is dominated by other labels, it can be removed from the label pool. The label setting algorithm is described as follows*.

**The Label Setting Algorithm**

***1*.** Create an initial label *L*_0_ = (0, 0, 0, …, 0;)

***2*.** *Set UTL*_0_ ≔ {*L*_0_} *and TTL*_0_ ≔ ∅.

***3*. For** *i* ∈ *T* **do**

***4*.**  set *UTL*_*i*_ ≔ ∅ and *TTL*_*i*_ ≔ ∅;

***5*. While** ⋃_*i*∈*T*_ *UTL*_*i*_ ≠ ∅ **do**

***6*.**  Choose a label *L*_*i*_ ∈ *UTL*_*i*_ and *UTL*_*i*_ ≠ ∅

***7*.**  **For all** (*i*, *j*) ∈ *A* **do**

***8*.**   Using extension functions, extend label *L*_*i*_ along arc (*i*, *j*) ∈ *A*

to create a label *L*_*j*_

***9*.**   **if** *L*_*j*_ satisfies constraints on task and shift **Then**

***10*.**    Set *UTL*_*j*_ ≔ *UTL*_*j*_ ⋃{*L*_*j*_}

***11*.**    Discard from the set *UTL*_*j*_ ⋃*TTL*_*j*_ the labels which are

dominated by the dominance rules

***12*.**   Set *UTL*_*i*_ ≔ *UTL*_*i*_ \ {*L*_*i*_} and *TTL*_*i*_ ≔ *TTL*_*i*_ ⋃{*L*_*i*_}

***13*.**  Find the label $L=\left(C,\;TI,\;SSQ,\;Spro,\;V^{t_{1}},\;\ldots ,\;V^{t_{N}}\right)$ with the

maximum *c*

*Let us define* *TTL*_*i*_ *and* *UTL*_*i*_ *to be the sets of treated and untreated labels of task* *i* ∈ *T*, *respectively. Step 1 to Step 4 describes the initialization of the algorithm. The main loop, starting from Step 5 to Step 12, deals with extending all the untreated non-dominated labels. If label* *L*_*i*_ *(with maximum* *reduced cost*) *is chosen in Step 6, it is extended along all the arcs* (*i*, *j*) ∈ *A* *to get label* *L*_*j*_. *If label* *L*_*j*_ *represents a feasible* 0 − *j* *path, set* *UTL*_*j*_ *will be updated; Step 11 invokes a dominance procedure to determine whether or not label* *L*_*j*_ *is dominated by other labels in the current set* *UTL*_*j*_⋃*TTL*_*j*_, *and whether or not it dominates other labels in this set. When the main loop is completed, if set* ⋃_*i* ∈ *T*_ *UTL*_*i*_ *equals to* ⌀, *a path with highest reduced cost is found in Step 13 by examining the labels from the set* *TTL*_*N* + 1_.

### 4.4 Accelerating strategy

For column generation algorithm, most of the computational time is spent on solving the sub-problem. Since any column with positive reduced cost helps in improving the objective function value of the RMP, the sub-problem need not be solved to optimality at every iteration. Heuristic techniques, as opposed to exact algorithms, are capable of quickly identifying high quality solutions. By this way, the exact algorithm can be integrated with the heuristic technique. While solving the sub-problem, a heuristic technique will be first applied to identify some high-quality columns with positive reduced costs. Upon no improvement in the objective value, the exact algorithm will be applied to further improve the solution quality or to validate the optimality of the current solution.

The following sections describe in detail the accelerating strategies proposed to expedite the column generation process.

1. *Initial columns*. To prevent slowing down the solution process of the sub-problem in the first iteration by very large dual values, the column generation process is started with a sub-set of all routes *P* in the restricted master problem that should be sufficient to obtain a feasible solution. The initial solution is constructed as follows: Select a shift, assign all the feasible tasks in the task pool to the shift; then, move to the next shift to repeat the assignment process with the left over tasks until all the shifts are examined.
2. *Heuristic label setting algorithm*. To accelerate the solution process of the RMP, Tabu Search (TS) is invoked prior to the application of the label setting algorithm to solve the sub-problem. The procedure starts with an initial solution and iteratively explores the neighborhood solutions according to some pre-designed operators. To avoid visiting the same solution again, a taboo list is maintained to keep track of all the recent moves that are forbidden for ‘*η*’ subsequent iterations (Tabu length). If a particular move results in a solution with an objective value better than that of the current best-known solution, the move is allowed even if it is in the taboo list (i.e., the aspiration criteria). Our proposed TS algorithm is similar to that of proposed by Dayarian et al. [43]. The seed solution is constructed using the basic variables of the RMP and the algorithm iteratively explores the neighborhood for better quality solutions. The total number of iterations is controlled by a pre-set parameter *MIter*. Upon convergence of the TS algorithm, the label setting algorithm is executed to either improve the quality of the solution further or establish the optimality of the current solution.

### 4.5 Branching

When branching is required to be performed at a node of the branch-and-bound search tree, branching decision can be imposed on the total number of shifts used by computing $S^{{}^{\prime }}=\sum_{s\in S}\;\;\sum_{r\in P_{s}}\;X_{r}^{s}$. If the value is fractional, two child nodes are created by performing dichotomic branching as follows:

1. $\sum_{s\in S}\;\;\sum_{r\in P_{s}}\;X_{r}^{s}\leq \lfloor S^{{}^{\prime }}\rfloor$ imposed on one child node;
2. $\sum_{s\in S}\;\;\sum_{r\in P_{s}}\;X_{r}^{s}\geq \lceil S^{{}^{\prime }}\rceil$ imposed on the other child node.

**Fig 1:**
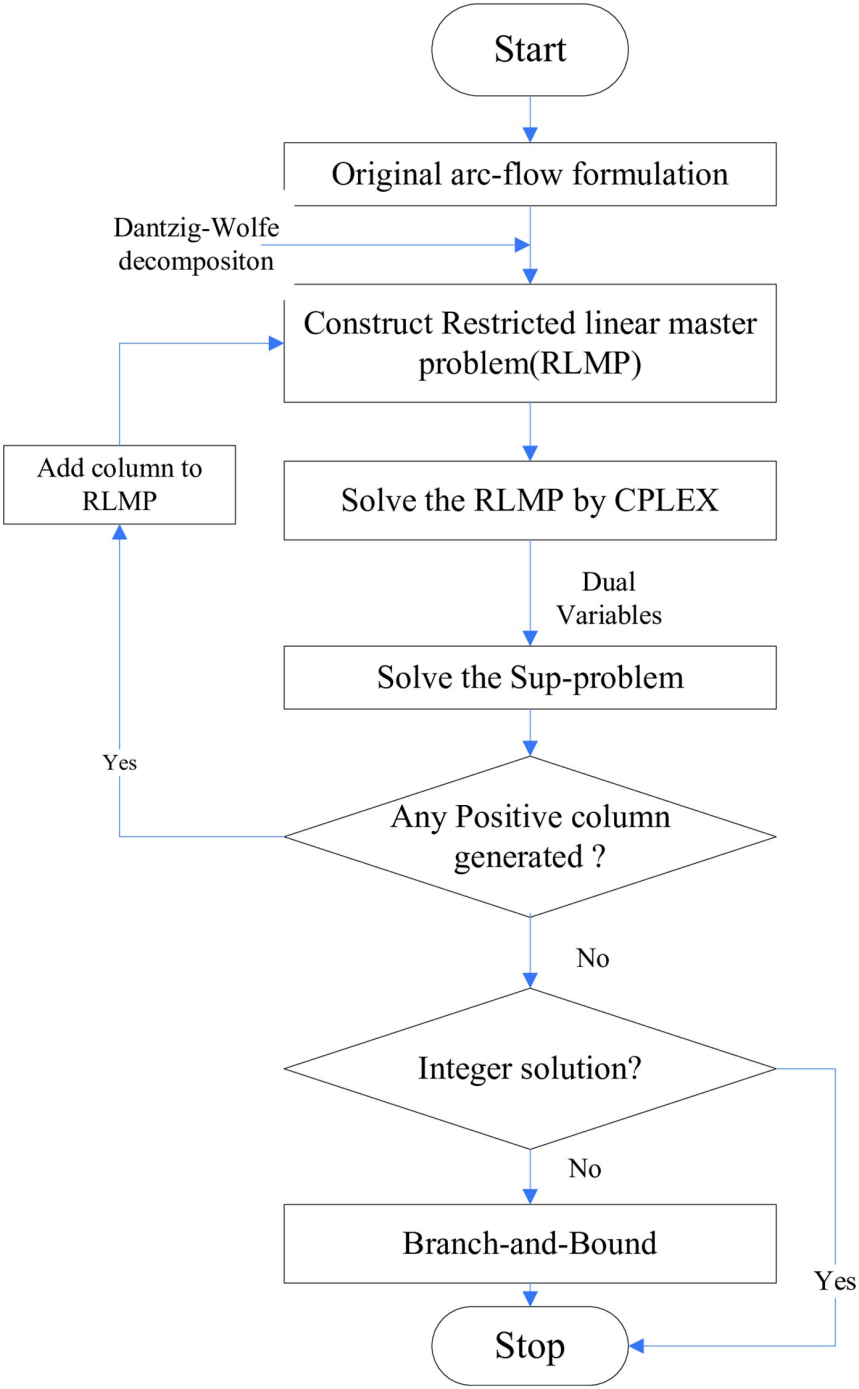
Flow chart of branch-and-price algorithm.

If *S*′ turns out to be an integer value, binary arc flow variables are branched. If fractional variables exist, typically, the branching is done on the one with its fractional part closest to 0.5. The search tree is explored using best-first and single arc branching strategies (Desrochers et al. [45]). The flowchart of the branch-and-price algorithm is described in Fig 1.

## 5 Computational experiments

### 5.1 Experimental setup

To assess the performance of the proposed methodology, we make use of real-life problem instances provided by the ground handling service provider. The data for “tasks” consists of task number, task type, task start time, task duration, task priority and task qualification/proficiency pair. Information pertaining to “shifts” consists of shift number, shift start time, shift duration and shift qualification/proficiency pair. Typically, for most of the problem instances, the number of total daily tasks is just over 330 while the available shifts is in the range of 100. Thus, the ratio of task numbers over shift numbers roughly equals to 3.3:1. Each problem instance is characterized by the number of tasks and the number of shifts (exclusive of their properties) and represented with the syntax Txx-sxx where T represents tasks and ‘s’ represents shifts. For example, T10-s3 represents 10 tasks that are to be assigned to 3 shifts.

All the computational experiments were performed on a desktop computer driven by an Intel Pentium dual-core processor of 2.8GHz speed and 4GB RAM with a computational time limit of 3600 seconds. The proposed branch-and-price algorithm was coded using Visual C++ and the problem instances of both the arc-flow formulation and the restricted master problems were solved to optimality by the IBM CPLEX 12.2 solver. Based on the preliminary experiments of TS, the parameters used in this paper were fixed as *η* = 10 and *MIter* = 50.

We shall provide brief description pertaining to different columns used in the following tables. The first column “Instance” represents the problem instances; column “CPLEX (Model_IP)” represents the computational results of arc-flow MIP formulation when solved by CPLEX; column “BP” represents the computational results pertaining to the branch-and-price algorithm with label setting algorithm for the sub-problem; column “BPT” represents the computational results of the branch-and-price algorithm with the proposed accelerating strategy. Columns “OPT” and “Time” report the optimal value and computational time (in seconds), respectively. Column “UB” reports the upper bound value at the root node; column “Node” gives the number of nodes generated in the branch-and-bound tree; column “Gap” gives the relative difference (in percentage) between UB and OPT, which is calculated as (UB-OPT)/UB × 100%; and column “TS” reports the time consumed by Tabu search algorithm.

### 5.2 Computational results of small-sized instances

The computational results of 11 small-sized problem instances, that satisfy the task to shift ratio of 3.3:1, are presented in Table 5. The results show that all the proposed methodologies i.e., CPLEX (Model_IP), BP and BPT can generate optimal solutions in a very short period of time. However, on an average, BPT consumes the least amount of time. A value of ‘1’ under the column “Node” indicates that the integer optimal solution is obtained at the root node itself without branching. For the BP algorithm, in 8 out of 11 instances, the integer optimal solutions are obtained at the root node. This value is even better for the BPT algorithm with 9 out of 11 instances. As far as the quality of bounds is concerned, the average and maximum % gaps of the BP and the BPT algorithms are 0.45, 2.46 and 0.30, 2.03, respectively. With respect to the computational time, the average computational time improves from 27.69s (BP algorithm) to 19.73s (BPT algorithm). The comparison clearly endorses the fact that BPT can accelerate the computational process of the sub-problem. Moreover, the identical optimal solutions reported by CPLEX (Model_IP) when compared against BP and BPT validates the proposed arc-flow formulation. To summarize, for small-sized problem instances, with computational time as the basis for comparison, CPLEX (Model_IP) is superior to the BP algorithm, while BPT algorithm outperforms both CPLEX (Model_IP) and the BP algorithm.

**Table 5:** Computational results for small-sized instances.

Instance	CPLEX(Model_IP)		BP					BPT					
	OPT	Time	UB	OPT	Node	Gap	Time	UB	OPT	TS	Node	Gap	Time
T10-s3	570	4.93	570	570	1	0	3.87	570	570	0.47	1	0	4.53
T13-s4	230	4.54	230	230	1	0	4.61	230	230	0.53	1	0	4.27
T17-s5	1170	5.62	1170	1170	1	0	7.06	1170	1170	0.81	1	0	5.18
T20-s6	920	11.63	920	920	1	0	10.59	920	920	0.79	1	0	6.93
T23-s7	1025	15.41	1025	1025	1	0	21.33	1025	1025	1.19	1	0	12.69
T26-s8	5030	27.03	5507.9	5030	3	0.95	47.66	5030	5030	1.44	1	0	20.41
T30-s9	3450	28.63	3450	3450	1	0	24.71	3450	3450	1.32	1	0	22.56
T33-s10	4760	30.12	4830.9	4760	3	1.49	50.07	4818.5	4760	3.54	3	1.23	39.81
T36-s11	5540	31.68	5540	5540	1	0	39.72	5540	5540	1.64	1	0	28.74
T40-s12	7890	37.48	8084.1	7890	3	2.46	54.82	8050.1	7890	6.27	3	2.03	40.65
T43-s13	4895	36.79	4895	4895	1	0	40.25	4895	4895	1.48	1	0	31.26
Average	3225.5	21.26	3293	3225.5	1.54	0.45	27.69	3245.3	3225.5	1.77	1.36	0.3	19.73

### 5.3 Computational results of large-sized instances

The computational results of CPLEX (Model_IP), BP and BPT for large-sized instances are presented in Table 6 It can be observed from the computational results that when the instance size increases to T150-s45, CPLEX (Model_IP) fails to converge to optimality within the stipulated time period. Therefore, for instances T150-s45 to T200-s60, the computational results pertaining to BP and BPT algorithms are presented. Beyond 200 tasks, since BP also fails to obtain an integer solution within 3600 seconds, the computational results of only BPT are provided. This implies that, on the basis of size, BPT is capable of solving large-sized instances and easily outperforms BP and CPLEX (Model_IP).

**Table 6:** Computational results for large-sized instances.

Instance	CPLEX(Model_IP)		BP					BPT					
	OPT	Time	UB	OPT	Node	Gap	Time	UB	OPT	TS	Node	Gap	Time
T50-s15	7420	78.7	7424.9	7420	3	0.84	101.37	7420	7420	1.27	1	0	37.68
T60-s18	14660	33.5	14660	14660	1	0	44.86	14660	14660	2.08	1	0	24.43
T70-s21	10605	73.05	10605	10605	1	0	41.25	10605	10605	1.63	1	0	19.12
T80-s24	11220	188.9	11484.7	11220	5	2.36	127.57	11220	11220	10.05	1	0	57.67
T90-s27	15210	2018.01	15813.8	15210	13	3.97	1254.82	15585.7	15210	8.36	5	2.47	394.26
T100-s30	16895	396.7	16895	16895	1	0	372.65	16895	16895	6.79	1	0	113.37
T110-s33	21620	2396.9	22136.7	21620	23	2.39	1281.02	22063.2	21620	4.29	7	2.05	471.16
T120-s36	22675	1890.7	23264.5	22675	11	2.6	1128.97	23112.6	22675	3.71	3	1.93	225.34
T130-s39	26320	2609.3	26320	26320	1	0	369.72	26320	26320	11.52	1	0	147.46
T140-s42	24095	3506.43	24820.2	24095	15	3.01	1421.45	24622.7	24095	3.68	3	2.19	350.79
T150-s45	/	/	31010.3	29675	9	4.5	1036.01	30686.9	29675	24.37	7	3.41	568.84
T160-s48	/	/	36430.4	35390	17	2.94	1594.87	36051.8	35390	19.15	5	1.87	507.19
T170-s51	/	/	37480.4	36185	33	3.58	2057.54	37158.4	36185	86.34	13	2.69	1421.02
T180-s54	/	/	33796.6	33160	6	1.92	979.26	33160	33160	7.69	1	0	204.73
T190-s57	/	/	35375.1	34425	45	2.76	2217.45	35089.4	34425	36.2	15	1.93	625.47
T200-s60	/	/	45785.5	43145	57	6.12	3583.41	44206.4	43145	187.05	19	2.46	1945.86
T210-s63	/	/	/	/	/	/	/	38516.6	36880	80.66	15	4.24	1497.25
T220-s66	/	/	/	/	/	/	/	39595.1	38145	314.38	29	3.67	2643.38
T230-s69	/	/	/	/	/	/	/	49505.4	46865	379.19	35	5.33	3287.69
T240-s72	/	/	/	/	/	/	/	50456.7	47940	405.27	41	4.99	3591.93

It can be observed from Table 6 that, for BPT algorithm, the maximum % gap reported is less than 5.5%. This underscores the high quality of upper bounds obtained at the root node by LP relaxation of the set partitioning formulation. While comparing BP and BPT algorithm on the basis of value of “node”, it is observed that the branching number is significantly smaller for BPT. This clearly highlights the computationally efficient design of BPT and the role of accelerating strategy, in particular.

### 5.4 Computational results of varied instance properties

Apart from the size of the problem, we intend to study the impact of changing other important characteristics such as the number of tasks, number of shifts, shift working time etc. on the objective function value and the computational time to optimality. Though the methodologies are capable of solving large problem instances, we choose to restrict the testbed to only small sized instances (reported in Table 5). In Table 7, we examine the impact of increasing the number of tasks while keeping the number of shifts constant on the objective function value. For example, for instances T10-s3, T11-s3 and T12-s3, the shifts are identical. As expected, all the three methodologies i.e., CPLEX (Model_IP), BP and BPT could solve the problem instances to optimality within very short computational times with BPT consuming the least average time. The results show that except for a few instances (T21-s6, T25-s8, T26-s8 and T27-s8), by and large, there was no change in the objective function value. Since shifts are limited in number compared to the tasks, and hence when the number of shifts is fixed, only increasing the number of tasks has little influence on the value of the objective function, nor can it significantly improve the utilization rate of shifts.

**Table 7:** The impact of more tasks.

Instance	CPLEX(Model_IP)		BP					BPT					
	OPT	Time	UB	OPT	Node	Gap	Time	UB	OPT	TS	Node	Gap	Time
T10-s3	570	4.93	570	570	1	0	3.87	570	570	0.47	1	0	4.53
T11-s3	570	5.79	570	570	1	0	3.94	570	570	0.44	1	0	4.68
T12-s3	570	4.89	570	570	1	0	3.45	570	570	0.41	1	0	4.49
T13-s4	230	4.54	230	230	1	0	4.61	230	230	0.53	1	0	4.27
T14-s4	230	5.02	230	230	1	0	6.04	230	230	0.51	1	0	4.45
T15-s4	230	6.35	230	230	1	0	8.21	230	230	0.94	1	0	5.34
T16-s5	1170	6.03	1170	1170	1	0	8.65	1170	1170	1.05	1	0	5.58
T17-s5	1170	5.62	1170	1170	1	0	7.06	1170	1170	0.81	1	0	5.18
T18-s5	1170	10.29	1170	1170	1	0	9.01	1170	1170	0.83	1	0	4.85
T19-s6	920	11.63	920	920	1	0	10.59	920	920	0.79	1	0	6.93
T20-s6	920	11.63	920	920	1	0	10.59	920	920	0.79	1	0	6.93
T21-s6	1520	18.42	1539.6	1520	3	1.29	29.67	1520	1520	0.72	1	0	9.82
T22-s7	1025	15.41	1025	1025	1	0	21.33	1025	1025	1.19	1	0	14.69
T23-s7	1025	15.41	1025	1025	1	0	21.33	1025	1025	1.19	1	0	12.69
T24-s7	1025	21.35	1025	1025	1	0	25.44	1025	1025	1.24	1	0	16.35
T25-s8	4835	24.95	4934.1	4835	3	2.05	50.68	4835	4835	1.28	1	0	24.63
T26-s8	5030	27.03	5507.9	5030	3	0.95	47.66	5129.1	5030	3.16	3	1.97	20.41
T27-s8	5050	25.06	5182.8	5050	3	2.63	47.23	5163.6	5050	3.22	3	2.25	30.25
T28-s9	3450	25.44	3450	3450	1	0	26.42	3450	3450	1.42	1	0	23.24
T29-s9	3450	26.01	3450	3450	1	0	24.59	3450	3450	1.33	1	0	22.45
T30-s9	3450	28.63	3450	3450	1	0	24.71	3450	3450	1.32	1	0	22.56
Average	1791	14.5	1825.7	1791	1.38	0.33	18.81	1801.1	1791	1.25	1.19	0.21	12.11

Similarly, in Table 8, we explore the impact of increasing the number of shifts while keeping the number of tasks constant on the objective function value. From the test results in the Table 8, it can be found that the average value of OPT is 2056.1, which is 14.80% more when compared with the OPT 1791.0 in Table 7. The results indicate that the increasing the number of shifts has a greater impact on the value of the objective function than increasing the number of tasks. In the actual data, the number of tasks is far greater than the number of shifts. When the number of shifts increases, more tasks will be executed, so the value of the objective function increases. Therefore, when the number of tasks is fixed, appropriately increasing the number of shifts can improve the execution rate of tasks.

**Table 8:** The impact of more shifts.

Instance	CPLEX(Model_IP)		BP					BPT					
	OPT	Time	UB	OPT	Node	Gap	Time	UB	OPT	TS	Node	Gap	Time
T10-s3	570	4.93	570	570	1	0	3.87	570	570	0.47	1	0	4.53
T10-s4	840	6.35	840	840	1	0	2.53	840	840	0.69	1	0	2.34
T12-s3	570	4.89	570	570	1	0	3.45	570	570	0.41	1	0	4.49
T12-s4	840	5.04	840	840	1	0	2.67	840	840	0.41	1	0	2.51
T13-s4	230	4.54	230	230	1	0	4.61	230	230	0.53	1	0	4.27
T13-s5	720	6.42	720	720	1	0	5.48	720	720	0.57	1	0	4.07
T15-s4	230	6.35	230	230	1	0	8.21	230	230	0.94	1	0	5.34
T15-s5	720	10.23	720	720	1	0	10.16	720	720	1.13	1	0	5.12
T16-s5	1170	6.03	1170	1170	1	0	8.65	1170	1170	1.05	1	0	5.58
T16-s6	1260	12.13	1260	1260	1	0	8.66	1260	1260	1.05	1	0	5.58
T18-s5	1170	10.29	1170	1170	1	0	9.01	1170	1170	0.83	1	0	4.85
T18-s6	1260	10.93	1260	1260	1	0	9.42	1260	1260	0.94	1	0	4.91
T19-s6	920	11.63	920	920	1	0	10.59	920	920	0.79	1	0	6.93
T19-s7	1580	11.7	1580	1580	1	0	10.59	1580	1580	0.79	1	0	6.93
T21-s6	1520	18.42	1539.6	1520	3	1.29	29.67	1520	1520	0.72	1	0	9.82
T21-s7	2180	13.86	2180	2180	1	0	11.46	2180	2180	1.24	1	0	10.45
T22-s7	1025	15.41	1025	1025	1	0	21.33	1025	1025	1.19	1	0	14.69
T22-s8	2080	19.53	2117.2	2080	3	1.79	40.48	2105.8	2080	4.05	3	1.24	31.58
T24-s7	1025	21.35	1025	1025	1	0	25.44	1025	1025	1.24	1	0	16.35
T24-s8	2080	19.57	2117.2	2080	3	1.79	40.51	2105.8	2080	4.1	3	1.24	31.61
T25-s8	4835	24.95	4934.1	4835	3	2.05	50.68	4835	4835	1.28	1	0	24.63
T25-s9	5420	58.36	5438.5	5240	5	3.79	64.37	5561.4	5420	1.28	3	2.61	36.87
T27-s8	5050	25.06	5182.8	5050	3	2.63	47.23	5163.6	5050	3.22	3	2.25	30.25
T27-s9	5635	30.17	5757.8	5635	3	2.18	59.41	5727.4	5635	4.51	3	1.64	38.79
T28-s9	3450	25.44	3450	3450	1	0	26.42	3450	3450	1.42	1	0	23.24
T28-s10	3870	31.2	3906.3	3870	3	0.94	52.88	3897.9	3870	3.48	3	0.72	36.48
T30-s9	3450	28.63	3450	3450	1	0	24.71	3450	3450	1.32	1	0	22.56
T30-s10	3870	32.31	3906.3	3870	3	0.94	52.96	3897.9	3870	3.54	3	0.72	36.62
Average	2056.1	16.99	2075.4	2049.6	1.71	0.62	23.05	2072.3	2056.1	1.54	1.5	0.37	15.41

Through the inspection of the actual data, it is found that the task duration of some tasks is 30 minutes (“TE-TS = 30”), and there are some shifts meet the requirements of qualification and proficiency, but these shifts do not have enough working hours (“SE-SS”), so these tasks cannot be completed. In the airport, with the difference of off-peak season and the occurrence of temporary emergencies, the task volume of the airport will increase sharply in a certain period of time, and it is very common for the ground staff to extend the working time of 30 minutes appropriately. Therefore, in Table 9, we study the impact of extending 30 minutes of the working time of a shift on the benefits derived. The test results indicate that the average value of OPT is 3361.8, which is 4.23% more when compared with no overtime, but the average computational time doesn’t change very much. This shows that appropriately increasing the working hours of shifts in special periods can improve the task completion rate and work efficiency of shifts, and significantly improve the value of objective function at the same time.

**Table 9:** The impact of increasing shift working time.

Instance	CPLEX(Model_IP)		BP					BPT					
	OPT	Time	UB	OPT	Node	Gap	Time	UB	OPT	TS	Node	Gap	Time
T10-s3	570	4.93	570	570	1	0	3.87	570	570	0.47	1	0	4.53
T13-s4	290	5.07	290	290	1	0	4.82	290	290	0.55	1	0	4.31
T17-s5	1670	7.89	1670	1670	1	0	7.25	1670	1670	0.94	1	0	6.04
T20-s6	1250	10.15	1250	1250	1	0	10.81	1250	1250	0.84	1	0	7.18
T23-s7	1025	15.41	1025	1025	1	0	21.33	1025	1025	1.19	1	0	12.69
T26-s8	5285	29.31	5765.9	5285	3	0.91	48.05	5285	5285	1.44	1	0	23.45
T30-s9	3450	28.63	3450	3450	1	0	24.71	3450	3450	1.32	1	0	22.56
T33-s10	4760	30.12	4830.9	4760	3	1.49	50.07	4818.5	4760	3.54	3	1.23	39.81
T36-s11	5600	32.81	5600	5600	1	0	37.19	5600	5600	1.81	1	0	29.42
T40-s12	7915	37.48	8102.6	7915	3	2.37	58.63	8069.3	7915	5.46	3	1.95	42.69
T43-s13	5165	36.97	5289.9	5165	3	2.42	56.94	5165	5165	3.87	1	0	32.58
Average	3361.8	21.71	3440.4	3361.8	1.73	0.65	29.42	3381.2	3361.8	1.95	1.36	0.29	20.48

Lastly, we seek to study the impact of qualification/proficiency of tasks on the OPT value. The problem instances in Table 10 are constructed by adapting the relevant instances in Table 5. For the two instances with the same number of shift, the first instance is constructed by reducing the qualification of 50% of the tasks (represented by T) and the second instance is constructed with reduced proficiency levels (represented by T’). According to the change of mean value in Table 10, it is found that reducing the requirement of qualification of a task will have a great impact on the objective function. The value of the objective function will increase by up to 20.82%, which will have a more intuitive impact on the completion rate of airport tasks. Therefore, when the shift is set, reducing the task qualification requirement can not only increase the completion rate of the task, so that the profit is greater, but also control the waste of resources.

**Table 10:** The impact of reducing task requirements.

Instance	CPLEX(Model_IP)		BP					BPT					
	OPT	Time	UB	OPT	Node	Gap	Time	UB	OPT	TS	Node	Gap	Time
T10-s3	1770	5.18	1770	1770	1	0	4.84	1770	1770	0.38	1	0	4.65
T’10-s3	570	4.93	570	570	1	0	3.87	570	570	0.47	1	0	4.53
T13-s4	530	4.93	530	530	1	0	4.14	530	530	0.35	1	0	4.86
T’13-s4	230	4.54	230	230	1	0	4.61	230	230	0.53	1	0	4.27
T17-s5	3570	8.67	3570	3570	1	0	8.81	3570	3570	1.23	1	0	6.25
T’17-s5	1170	5.62	1170	1170	1	0	7.06	1170	1170	0.81	1	0	5.18
T20-s6	1580	12.43	1580	1580	1	0	10.67	1580	1580	1.02	1	0	9.37
T’20-s6	920	11.63	920	920	1	0	10.59	920	920	0.79	1	0	6.93
T23-s7	2020	17.08	2020	2020	1	0	22.42	2020	2020	1.25	1	0	18.94
T’23-s7	1025	15.41	1025	1025	1	0	21.33	1025	1025	1.19	1	0	12.69
T26-s8	5815	28.24	5895.8	5815	3	1.39	47.82	5815	5815	1.62	1	0	26.74
T’26-s8	5030	27.03	5507.9	5030	3	0.95	47.66	5030	5030	1.44	1	0	20.41
T30-s9	4680	31.47	4680	4680	1	0	35.59	4680	4680	1.57	1	0	21.62
T’30-s9	3450	28.63	3450	3450	1	0	24.71	3450	3450	1.32	1	0	22.56
T33-s10	5885	36.68	5957.3	5885	3	1.23	54.71	5935	5885	3.68	3	0.85	43.41
T’33-s10	4760	30.12	4830.9	4760	3	1.49	50.07	4818.5	4760	3.54	3	1.23	39.81
T36-s11	8760	67.25	9036.8	8760	5	3.16	89.25	8984.2	8760	7.24	3	2.56	49.26
T’36-s11	5540	31.68	5540	5540	1	0	39.72	5540	5540	1.64	1	0	28.74
T40-s12	9925	56.79	10147.3	9925	3	2.24	62.75	10097.7	9925	5.79	3	1.74	42.59
T’40-s12	7890	37.48	8084.1	7890	3	2.46	54.82	8050.1	7890	6.27	3	2.03	40.65
T43-s13	5720	38.47	5720	5720	1	0	45.46	5720	5720	2.06	1	0	32.43
T’43-s13	4895	36.79	4895	4895	1	0	40.25	4895	4895	1.48	1	0	31.26
Average	3897	24.59	3960.5	3897	1.73	0.59	31.42	3927.3	3897	2.08	1.45	0.38	21.69

## 6 Conclusions

This paper studies an airport ground service task assignment problem. The problem is modeled as a network optimization problem with mathematical formulation. Using Dantzig-Wolfe decomposition methodology, the model is separated into a master problem and a sub-problem. To solve the problem instances to optimality, a branch-and-price algorithm is proposed. For expediting the computational process, a Tabu Search (TS) algorithm is embedded into the branch-and-price algorithm to obtain some high-quality columns with positive *reduced costs*. The computational results show that the proposed branch-and-price algorithm with acceleration strategy (BPT) outperforms both the conventional IP model and the stand-alone branch-and-price technique in terms of computational time as well as size of the problem instances. The proposed acceleration strategy proved to be quite efficient in reducing the computational time. The proposed BPT is capable of solving problem instances with up to 240 tasks and 72 shifts in less than 3600 seconds.

Further research can consider different objective functions. When assigning a task, it is desired that, the shift proficiency can be as close as required, under the circumstance that the qualification requirement is met. This consideration can be included in the objective function. Moreover, partial task coverage can be explored, which allows a task to start earlier/later within the task duration as long as the minimal percentage of duration can be covered by the labor force.

Though this work considers flight schedules as deterministic in nature, practically, there is always some volatility associated with actual arrival and departure times. Developing a stochastic model that accommodates uncertainties can be a good extension of this work.

## Supporting information

S1 Data (ZIP)
